# Driving variables to explain soil organic carbon dynamics: páramo highlands of the Ecuadorian Real mountain range

**DOI:** 10.1007/s11368-025-04017-7

**Published:** 2025-04-08

**Authors:** Andrés A. Beltrán-Dávalos, Johanna Elizabeth Ayala Izurieta, Magdy Echeverría, Carlos Arturo Jara Santillán, Jochem Verrelst, Jesús Delegido, Agustín Merino, X. L. Otero

**Affiliations:** 1 Departament of Soil Science and Agricultural Chemistry, Unit for Sustainable Environmental and Forest Management, University of Santiago de Compostela, 27002 Lugo, Spain; 2 Group of Research for Watershed Sustainability (GISOCH), Faculty of Sciences, Escuela Superior Politécnica de Chimborazo (ESPOCH), Riobamba, 060155 Ecuador; 3 Faculty of Sciences, Escuela Superior Politécnica de Chimborazo (ESPOCH), Riobamba, 060155 Ecuador; 4 Faculty of Natural Resources, Escuela Superior Politécnica de Chimborazo (ESPOCH), Riobamba, 060155 Ecuador; 5 Image Processing Laboratory (IPL), University of Valencia, 46980 Paterna, Spain; 6 Departamento de Edafoloxía E Química Agrícola, Facultade de Bioloxía, CRETUS, Universidad de Santiago de Compostela, 15782 Santiago de Compostela, Spain

**Keywords:** Andean soils, CART regression trees, Highlands, Spatial variability

## Abstract

**Purpose:**

Large soil organic carbon (SOC) reserves and a high soil capacity for SOC storage within an ecosystem contribute to mitigating the release of carbon into the atmosphere. Developing new spatially-explicit SOC estimation methods at local and micro-watershed scales is essential for gaining landscape understanding of SOC variability.

**Methods:**

This study provides new insights into the spatial variability of SOC in the Andean páramo soils. A range of variables from different sources (i.e., geophysical, meteorological, topographic, and spectral) were analyzed to identify driving variables to explain the SOC dynamic in the Andean páramo highlands of the Real range in the central region of Ecuador. This information was used to calibrate a SOC prediction model using Classification and Regression Trees (CART) and soil data samples from the 0–30 cm soil horizon.

**Results:**

Eight key variables linking with the SOC storage were used to calibrate the model for SOC estimation with an accuracy of 67% with an RMSE value of 2.17%. Results reveal that sand content emerged as the most significant variable, while taxonomic suborder and protected area variables provided crucial supplementary information. This study improves the ability to detect changes in SOC, particularly in smaller areas where traditional predictors, often more suitable for regional or national assessments, may exhibit insufficient explanatory power.

**Conclusion:**

The Andean páramo highlands of the Real range show high capacity for storing SOC, with values ranging from 3.5% to 19%. This variability highlights the ecosystem's importance as a globally relevant carbon reservoir.

## Introduction

Soil is one of the most important reservoirs of carbon on Earth (IPCC 2022). The Intergovernmental Panel on Climate Change (IPCC) estimated the cumulative soil organic carbon (SOC) stock in the top meter of soil at 1502 Pg (Stockmann et al. 2013; IPCC 2022). In contrast, global estimates derived from the Harmonized World Soil Database (HWSD) suggest that approximately 1417 Pg of SOC are stored in the top meter of soil and approximately 716 Pg of SOC are in the top 0–30 cm (Pennock and McKenzie 2016).

The capacity of soils to store carbon depends on multiple properties and processes, such as soil composition (e.g., allophane and nutrient content), recalcitrance of soil organic matter (SOM), and edaphic (e.g., soil redox and acid–base) and climatic conditions (e.g. temperature, precipitation, etc.) (Van Breemen and Buurman 2002; Galicia et al. 2016; Yando et al. 2016). All these factors affect the rate of SOM mineralization mainly because they directly influence the soil microbial activity (Van Breemen and Buurman 2002; Mäkipää et al. 2023). Carbon is the main component of SOM, around 58% (Pribyl 2010). Regarding the main causes of soil transitioning from a carbon sink to a significant source of greenhouse gases, such as methane and NO_x_, are attributed to land-use change, deforestation, biomass burning, the conversion of natural ecosystems to agricultural land, wetland drainage, and soil cultivation (Lal 2004; Veber et al. 2018; Soleimani et al. 2019). All of them cause discharges of carbon into the atmosphere. According to Lal (2004) the depletion of the global SOC pool has contributed 78 ± 12 Pg of C to the atmosphere.

Andean páramo soils are characterized by a substantial carbon stock (50–205 Mg C ha^−1^ in the top 30 cm) that is strongly related to lithology and soil taxonomy, where Andosols stand out with the higher amounts of SOM (Ayala Izurieta et al. 2021; Beltrán-Dávalos et al. 2022). Andosols are the predominant soil type in the páramo region. In these dark soils, SOM forms stable complexes with non-crystalline soil components. As a result, the decomposition of SOM is significantly delayed, leading to the accumulation of SOC (Buytaert et al. 2005). This underlines the importance of understanding the dynamics of SOC in ecosystems with high SOC storage capacity, with emphasis on ecosystems with a particular lithology and taxonomy, such as the páramo.

Soil carbon storage is regulated by both soil processes and the ecosystem’s climatic conditions (Galicia et al. 2016; Yando et al. 2016). In high-altitude ecosystems, characterized by lower temperatures, the rate of SOM decomposition is significantly reduced (Pinos-Morocho et al. 2021). While abundant SOM can enhance soil fertility, it can also increase the risk of its rapid mineralization to CO_2_ following disturbances. This mineralization process releases inorganic nutrients such as nitrogen and phosphorus, which are often limiting elements in soil fertility (Du et al. 2020). Furthermore, microbial activity plays a crucial role in carbon mineralization, leading to the production of CO_2_ that can either be released into the atmosphere or incorporated into microbial biomass.

In this context, SOC storage in an ecosystem (Cerón Rincón and Aristizábal Gutiérrez 2012; Borowik and Wyszkowska 2016) and the capacity of soils to store SOC have significant implications for climate regulation, prevention of erosion, and nutrient storage, recycling, processing, and acquisition (Wiesmeier et al. 2019).

In view of the above, a global effort is being undertaken to better understand the mechanisms of SOM stabilization, for which it is essential to know the parameters that determine its spatial variability (Li et al. 2023; Mäkipää et al. 2023; Xiao et al. 2023). The Cordillera Real of Ecuador, known as the Eastern Cordillera, is located in the tropical Andes region, and therefore has an impact on regional and global climate regulation (Rabatel et al. 2013), i.e., high elevations and glaciers help to maintain climate balance by reflecting solar radiation and cooling the atmosphere. Its topography acts as a natural barrier against extreme weather events, such as storms and cold fronts, making this páramo a region of geological, biological, climatological, and glaciological interest. Therefore, to ensure the sustainability of their ecosystem services while maintaining and enhancing the multiple benefits they provide, both locally and regionally it is essential to conserve high Andean ecological systems. In terms of local anthropogenic activities, intensive cattle ranching and the burning of endemic vegetation to replace it with other vegetation are common among villagers and could be considered a threat to carbon stocks (Berenguer et al. 2014). At present, these impacts affect small areas, but they are expected to increase in the coming years due to the demand for more agricultural land and expansion in anthropogenic activities.

The protection of páramo and its vegetation (i.e., *Calamagrostis intermedia, Calamagrostis effuse, Azorella spp.,* and *Plantago rigida*) is achieved by delimiting protected areas, promoting practices for the sustainable use of natural resources, and engaging local communities in the decision-making and overseeing of these spaces. Detailed information on SOC distribution is required to determine the appropriate use of hydrological resources, make informed decisions, and plan mitigation and adaptation actions, since areas with high SOC storage would be more sensitive to variations in rainfall intensity and slope gradients (Gao et al. 2018). Despite the interest and need for preserving areas with a high SOC percentage, such as high Andean regions, these areas are hardly studied, which could be explained by their harsh climatic conditions, with low temperatures, and their complex topography, with limited accessibility.

Modeling to estimate SOC storage capacity is a useful tool for promoting soil conservation and sustainable management within ecosystems. The prediction of SOC allows assessment of the impact of different forestry and agricultural practices on carbon reserves (Mandal et al. 2022). Regarding SOC estimation methods, the relationship between SOC and environmental variables is not explicit. Moreover, in complex areas, where relationships are inherently nonlinear, the application of nonlinear and nonparametric regression algorithms is essential. These algorithms establish functions concerning a group of independent variables and the associated variable, for which a learning phase based on training data is required (Verrelst et al. 2019). A diversity of regression algorithms for SOC mapping were assessed in earlier studies on highland soils, such as decision trees (Random Forest (Breiman 2001)) and kernel-based regression methods, such as K-nearest neighbors regression (Kramer 2013), kernel ridge regression (Suykens and Vandewalle 1999), kernel-based variational Heteroscedastic Gaussian Processes Regression method (Lázaro-Gredilla et al. 2014), and Gaussian processes regression (Rasmussen and Williams 2006), were assessed in earlier studies on highlands soils, where Random Forest excelled for topsoil SOC estimation (Ayala Izurieta et al. 2021, Ayala Izurieta et al. 2022). Random Forest is an ensemble of classification and regression trees (CART) (Breiman et al. 1984), and it has demonstrated a competitive performance compared with other machine learning algorithms such as support vector machines (Hearst et al. 1998), among others, in SOC studies under different weather conditions (Keskin et al. 2019; John et al. 2020).

A large number of variables have been assessed to study the SOC storage dynamics, including meteorological, topographic, geoinformation, and remote sensing variables. Regarding remote sensing variables, the 705 nm band of Sentinel 2 (S2) and SeLI index (Pasqualotto et al. 2019) have been demonstrated to be useful to explain SOC variations (Ayala Izurieta et al. 2022). Identifying key variables related to SOC dynamics could offer a compelling possibility to calibrate SOC prediction models, which are needed to quantify the spatial distribution of SOC in detail.

The spatially explicit detection of high SOC reserves helps to design sustainable strategies that promote the accumulation of organic carbon, such as the application of conservation agriculture techniques, crop rotation, use of organic fertilizers, and adequate waste management. All these activities play a role in preventing future ecosystem damage. Consequently, by comparing the predicted data with the actual measurements, it is possible to assess the impact of management and conservation practices on SOC content, thus providing information to modify and improve conservation actions. For instance, alternative methods that minimize the need for intensive in-situ sampling can be explored, with the potential not only to understand wetland SOC but also to minimize environmental impact.

In this context, earlier studies on Herbaceous paramo in the center zone of Ecuador have identified key variables for SOC mapping and estimation (Ayala Izurieta et al. 2021, 2022). This research introduces a novel approach by aiming to explain spatial variations in SOC while focusing on smaller-scale areas, such as micro-watersheds. The results can be analyzed independently for different purposes, such as the assessment of environmental impacts due to natural and anthropogenic factors, to reduce uncertainty in our understanding of global SOC reserves. This implies a challenge for existing models, as previously identified variables may not be sufficient on their own, given their potential lack of heterogeneity at such fine scales, thus complicating model calibration for SOC estimation. This research addresses this specific knowledge gap by developing a regression model that combines in situ soil sampling with topographic (i.e., elevation, erosion factor based on topography, slope, flow direction, and flow accumulation), meteorological (i.e., precipitation and temperature), geophysical (i.e., soil texture, sand, silt, erosion factor based on soil composition, geological unit, and taxonomy suborder of soil), and satellite data (i.e., spectral bands from S2, spectral indices using S2 bands, and biophysical variables from S2), as well as geoinformation related to protected areas. This study was conducted in two micro-watersheds in the paramo highlands of Cordillera Real in the central region of Ecuador, and the specific goals were: (i) to identify climatic and soil variables linked to SOC sequestration to calibrate a prediction model for SOC sequestration at the top 0–30 cm soil layer; and (ii) to spatially predict and map SOC in the soil profile 0–30 cm in the study area.

## Materials and methods

### Study area

**Fig. 1:**
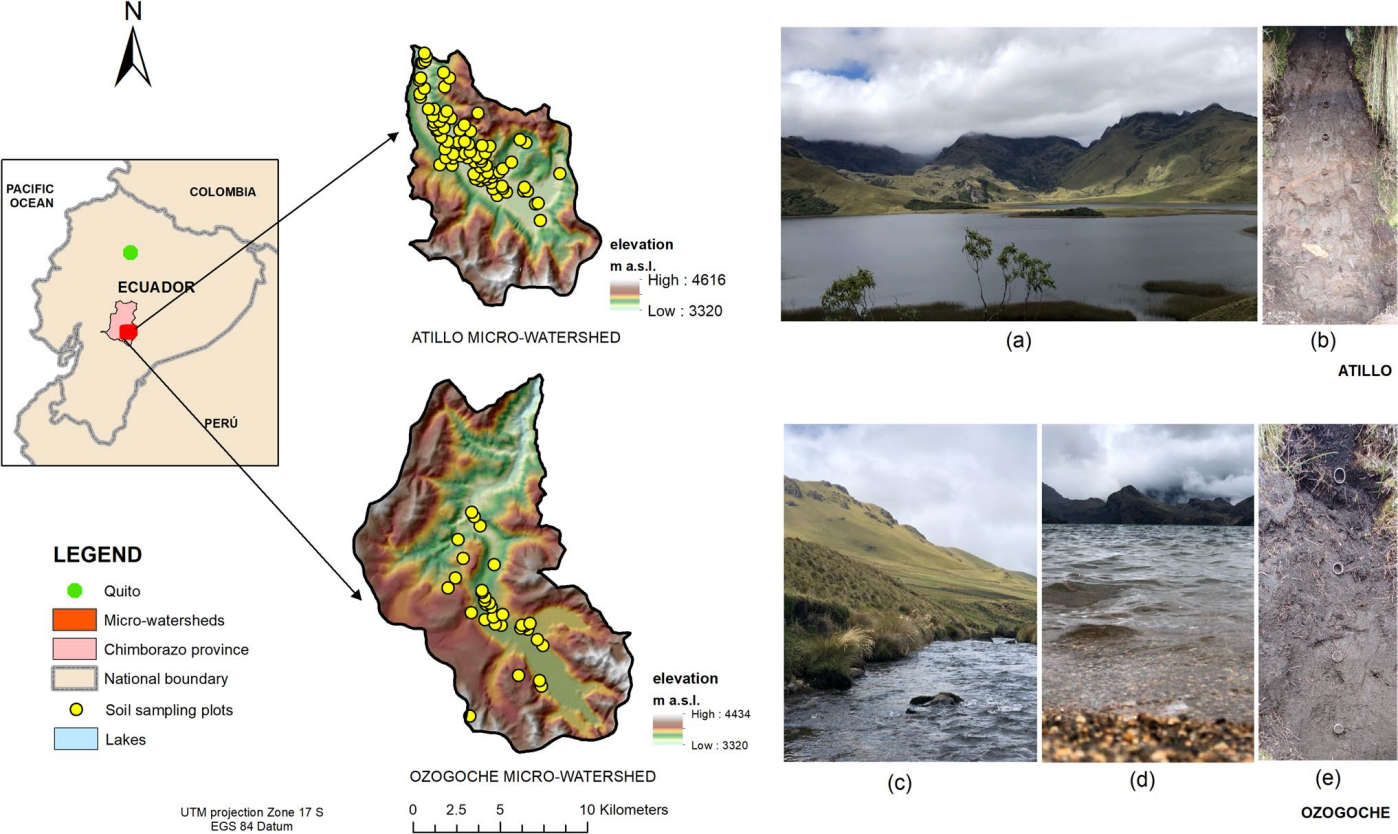
Study area: Atillo and Ozogoche micro-watersheds showing the locations of soil sampling points. **a** Atillo lacustrine ecosystem, (**b**) soil profile of Atillo, (**c**) and (**d**) Ozogoche lacustrine ecosystem and (**e**) soil profile of Ozogoche

The study area comprised the Atillo and Ozogoche micro-watersheds, located over the Real mountain range, in the center region of Ecuador (Fig. 1). Known locally as Cordillera Real, is the oldest range according to its geology. In Ecuador, the páramo highlands of the Cordillera Real vary based on their geology and geomorphology. Their main ecosystem services are hydrological regulation and supply. By supplying water to rivers and watersheds, mountain landscapes with peaks, valleys, lakes, lagoons, and glaciers play a crucial role in shaping the hydrological cycle (Mosquera et al. 2024). Both micro-watersheds cover an area of around 29,427 ha. They are located approximately 50 km southeast of Riobamba city in Ecuador.

Atillo extends over the Cordillera Real range, with its center located at 78°32′35″ W and 2°11′19'' S. Ozogoche is located at 78°35′18″ W and 2°15′18″ S (Fig. 1). Soil types mostly correspond to volcanic ash from the eruptive processes of Sangay volcano in the Chimborazo and Cotopaxi provinces. Based on soil taxonomy (USDA and NRCS 2014), Andosols, followed by Inceptisols, Mollisols, and Entisols, are the predominant soil types (Ministerio de Agricultura y Ganadería-MAG 2023) (Figs. 1b and e). The study area is surrounded by wetlands, lake systems, and grasslands in the upper area of Sangay National Park, which is designated a World Heritage Site since 1983 (Studholme et al. 2014).

The Atillo and Ozogoche micro-watersheds have a wide variety of aquatic ecosystems over relatively short distances (Steinitz-Kannan et al. 2020), including more than 45 lake systems with oligotrophic white waters, each with macrophyte species such as: *Spermatophyta, Charophyta,* and *C.demersum* (Steinitz-Kannan et al. 2020). The geomorphology of the highest Andean páramo lands plays a significant role in local weather patterns and in the shaping of diverse ecosystems such as: Herbaceous páramo, Evergreen Shrubland of Herbaceus páramo, Subnival Evergreen Shrubland of páramo, Subnival Ultra-humid Grassland of páramo, and Evergreen Forest of the páramo (Morocho and Chuncho 2019). These ecosystems are distributed from flat reliefs (Atillo periglacial valley) to strongly rugged reliefs (Ozogoche hills) (Figs. 1a, c and d). The presence of endemic forests is minimal and covers less than 4% of the area of the two micro-watersheds; the predominant species is *Polylepis racemosa* Ruiz & Pav*.* (GAD provincia de Chimborazo 2015).

Regarding climatic conditions, the altitude of the Atillo and Ozogoche micro-watersheds ranges between 3348 and 4150 m a.s.l.; therefore, the climate is cold, typical of highland areas, with an average annual temperature ranging from 5 to 8 °C. Annual mean precipitation is around 1659 mm (CONAE; Funk et al. 2014). Seasonal changes are not as marked as in other latitudes (Steinitz-Kannan et al. 2020), but a rainy season is identified from November to April (Buytaert et al. 2006). According to the Köppen-Geiger climate classification, the study area is characterized by a Cwb climate type, categorized as a Subtropical Highland climate (Peel et al. 2007; Crespo et al. 2011). Given their high elevation and latitude close to the equatorial line, the intensity of solar radiation is notably high (with a global horizontal irradiation ranging from 3.82 to 4.84 kWh/m^2^) (World Bank Group 2024).

Indigenous communities live in the Atillo and Ozogoche micro-watersheds, with an approximate population of 480 and 529 inhabitants, respectively (INEC 2010; Ferreira-Salazar et al. 2013). Due to mountainous climatic conditions, agricultural activity is limited above 3000 m a.s.l. Therefore, inhabitants are dedicated mainly to livestock farming activities and to the cultivation of exotic tree species, mainly *Pinus radiata D. Don* (Huber and Trecaman 2004). This activity is developed on hillside areas with slopes of approximately 5º.

### Soil data sampling

SOC in topsoil layers tends to be higher than in deeper soil layers, which can be attributed to increased carbon cycling and sequestration processes (Baisden and Parfitt 2007). While the greater potential for SOC accumulation in deeper soil layers is attributed to slower decomposition rates compared with surface soils (Ghimire et al. 2024). Moreover, studies in the páramo zone of Ecuador have reported SOC storage values up to 220.6 Mg C ha^−1^ in the 0–30 cm soil profile and up to 178.2 Mg C ha^−1^ in the 30–60 cm soil profile (Ayala Izurieta et al. 2022). Therefore, the capacity for SOC storage remains substantial in both soil profiles in the highlands, despite a slight decrease in SOC with increasing depth. Hence, this research focused on the 0–30 cm soil profile owing to its high capacity for SOC storage while concurrently exhibiting vulnerability to environmental damage. This vulnerability arises from the susceptibility of this layer to anthropogenic activities and to climate change. The implementation of effective strategies for monitoring and protecting these ecosystems is crucial for local governments.

A systematic soil sampling was planned as a reference for the Atillo and Ozogoche micro-watersheds. Next, a simple sampling was conducted in situ, for which sample plots were selected randomly after analyzing access. This consideration was necessary due to potential access limitations within specific areas of both the mountain and the valley micro-watersheds (Mendoza and Espinoza 2017). Sampling was designed, considering geopedological units and taxonomic suborders. Soil sampling in Atillo was conducted in April 2021, whereas the sampling in Ozogoche took place in May 2021, and February, November, and December 2022. A total of 113 composite samples made up of four random sub-samples (spaced 10 m apart) were obtained from the top 0–30 cm of soil (Mendoza and Espinoza 2017) distributed 80 in Atillo and 33 in Ozogoche micro-watersheds (Fig. 1).

The sampling plots were identified in situ with a Garmin Oregon 75t GPS using UTM coordinates. Before soil sample collection, plant debris and the O horizon were removed. Approximately 1 kg of soil was collected from the 0–30 cm soil profile using a blasthole to obtain SOC %. Additional soil samples were collected using stainless steel cylinders (Kopecky ring) of 48 mm in diameter and 105 cm^3^ in volume, which were used to determine bulk density (Agostini et al. 2014). Finally, the composite samples were geocoded by stratum identifier and sample number.

### Soil analysis

Each soil sample was homogenized by manual mixing and subsequently processed to measure moisture, bulk density, and organic carbon. Soil moisture content was measured by the gravimetric method. Samples were completely dried in an ESCO Isotherm oven at 105 °C for a 24-h period, following standard procedures outlined in the ISO 11465 standard. Bulk density was calculated by dividing the dry weight of the samples by their volume. Intact soil samples were collected using a Kopecky ring of known volume (approximately 105 cm^3^). Subsequently, samples were oven-dried at 105 °C for 48 h to remove residual moisture (Agostini et al. 2014). Regarding the SOC content, the organic carbon was determined using a CHN analyzer (“Thermo Scientific™ FLASH 2000 CHNS/O Analyzer”) on approximately 10 mg of each soil sample sieved through a 180 µm mesh. From the obtained organic carbon weight % and soil bulk density values, SOC contents were calculated in Mg C ha^−1^ (Eq. 1) (Lee et al. 2009).

1 $${SOC}_{{Mg C ha}^{-1}} ={SOC}_{\%}\times {Bulk Desity}_{g {cm}^{-3}}\times {Depth}_{cm}$$

### Satellite data and geoinformation assessment

To identify adequate driving variables to explain the spatial variability of SOC (SOC predictors) for the study area, we assessed 43 variables from different sources (Table 2).

First, we used variables derived from Sentinel-2 (S2) satellite imagery. S2 satellite data was selected given their high spatial resolution of S2, up to 10 m in specific spectral bands (B2-490 nm, B3-560 nm, B4-665 nm, B8-842 nm), their temporal resolution of five days with both S2 satellites (S2-A and S2B), and their free access (ESA 2015). Considering the sampling dates, the image used corresponded to the 17MQT tile from December 7th, 2022 (ID: S2A_MSIL2A_20221227T153621, tile extent: 100 km, cloud cover percentage within the scene: 33.8%, cloud cover percentage within the study area: 0%, Projection: UTM Zone 17S-Datum WGS84). This product has a processing Level 2A (i.e., per-pixel Bottom Of Atmosphere (BOA) reflectance). Equatorial highlands are characterized by frequent cloud cover over the scenes, however, cloud cover did not affect the study zone.

Image processing was conducted with the Sentinel Applications Platform (SNAP) software version 11.0 (ESA 2024). A total of 29 variables from S2 were derived, 13 variables corresponding to S2 spectral bands, 11 spectral indices, and 5 biophysical variables obtained from an automatic process in SNAP. The selection of these variables was based on the fact that spectral data variables such as multispectral bands and derived indices would help to explain and assess SOC reserves based on their usefulness for characterizing vegetation density and biomass (Wang et al. 2020). For instance, band 6 (740 nm) can be related to health and vegetation status (Wesemael et al. 2023). S2-dervived products such as spectral indices and biophysical variables were included to focus on surface characteristics in terms of vegetation cover, land use, and their changes, which are related to underground soil properties (Ayala Izurieta et al. 2021). The Normalized Difference Vegetation Index (NDVI) (Rouse et al. 1974), can serve as an adequate indicator of SOC due to the importance of vegetation as a driver of SOM accumulation (Yang et al. 2016; Wang et al. 2018). The Soil-Adjusted Vegetation Index (SAVI) minimizes the effect of soil brightness over the red and near-red bands with a correction factor (L) (Huete 1988) and can serve as an indicator for predicting topsoil SOC (Wang et al. 2018).

An additional 14 variables were analyzed to incorporate topographic, climatic, and geopedological information to identify SOC predictors and to obtain as much variability as possible. The study area exhibits uniform characteristics (based on its geology and soil taxonomy), which makes it difficult to explain changes in SOC storage. For this reason, we assessed variables at more local scales as SOC predictors. Topographic changes based on slope and its erosion effects were assessed based on a Digital Elevation Model (DEM) with a spatial resolution of 30 m, obtained from the Ecuadorian National information system (SNI 2011). The climatic variables precipitation and temperature were also included in the analysis due to their significant influence on ecosystem processes and their potential impact on SOC variability (Hijmans et al. 2005). Regarding granulometric fractions in soils**,** silt and clay contents stimulate the accumulation and stabilization of SOC, mainly composed of fine particles from silt and clays (Rietra et al. 2021). In addition, mineral characteristics of soils, such as Fe and Al content, pH values, presence of pedogenic oxides, and weather conditions (Ortner et al. 2022), can affect the soil's capacity to store or sequester SOC. This is due to a positive correlation found between SOC and total nitrogen, clay, silt, phosphorus, and potassium (Jakšić et al. 2021). Therefore, soil composition characteristics were assessed based on the national cartography developed by the Ministerio de Agricultura y Ganadería (MAG 2023).

### CART algorithm to predict SOC

With the aim of identifying driving variables that determine spatial changes in SOC and considering previous studies (Section 1), this study used the principles of the Random Forest method. Specifically, multiple CART regression models (Table 1) were developed and calibrated. This approach involved creating learning trees to closely observe the dynamics of the variables, thereby enhancing the interpretability of results. The complexity of the study area, due to its limited access and harsh challenging weather conditions, among other factors, makes SOC estimation challenging. Furthermore, while the study area is smaller with respect to studies at a regional scale, this introduced an additional complexity factor by decreasing the heterogeneity of some variables with potential as SOC predictors.

**Table 1:** Mathematical expression of the CART method (Breiman et al. 1984; Han et al. 2018)

Mathematic expression	Detail
Workspace to develop CART: $D=\left\{\left({x}_{11, }{x}_{12},{x}_{13,\dots .}{x}_{1n,}{y}_{1}\right),\left({x}_{21, }{x}_{22},{x}_{23,\dots .}{x}_{2n,}{z}_{2}\right),\dots . \left({x}_{m1, }{x}_{m2},{x}_{m3,\dots .}{x}_{mn,}{y}_{m}\right)\right\}$	$D$: input data matrix for training ${\left(x\right)}_{1}^{n}$: input variables $y$: output variable for a training dataset $m$: SOC samples
Binary recursive process to split the current space into two non-homogeneous subspaces. Residual variance to detect the best splitting variable and splitting point: ${Var}_{(j,s)}={min}_{S}\left\{{min}_{{s}_{1}\left(j,s\right)}\left[\sum_{{x}_{i}\in {S}_{1}\left(j,s\right)}{\left({y}_{i}-\overline{{y}_{1}}\right)}^{2}\right]+{min}_{{s}_{2}\left(j,s\right)}\left[\sum_{{x}_{i}\in {S}_{2}\left(j,s\right)}{\left({y}_{i}-\overline{{y}_{2}}\right)}^{2}\right]\right\}$	$S$: current space (parent node) ${\left(S\right)}_{1}^{2}$: subspaces (child nodes) $\overline{y:}$ output estimated variable from each subspace $j$: split variable $s$: split point

**Fig. 2:**
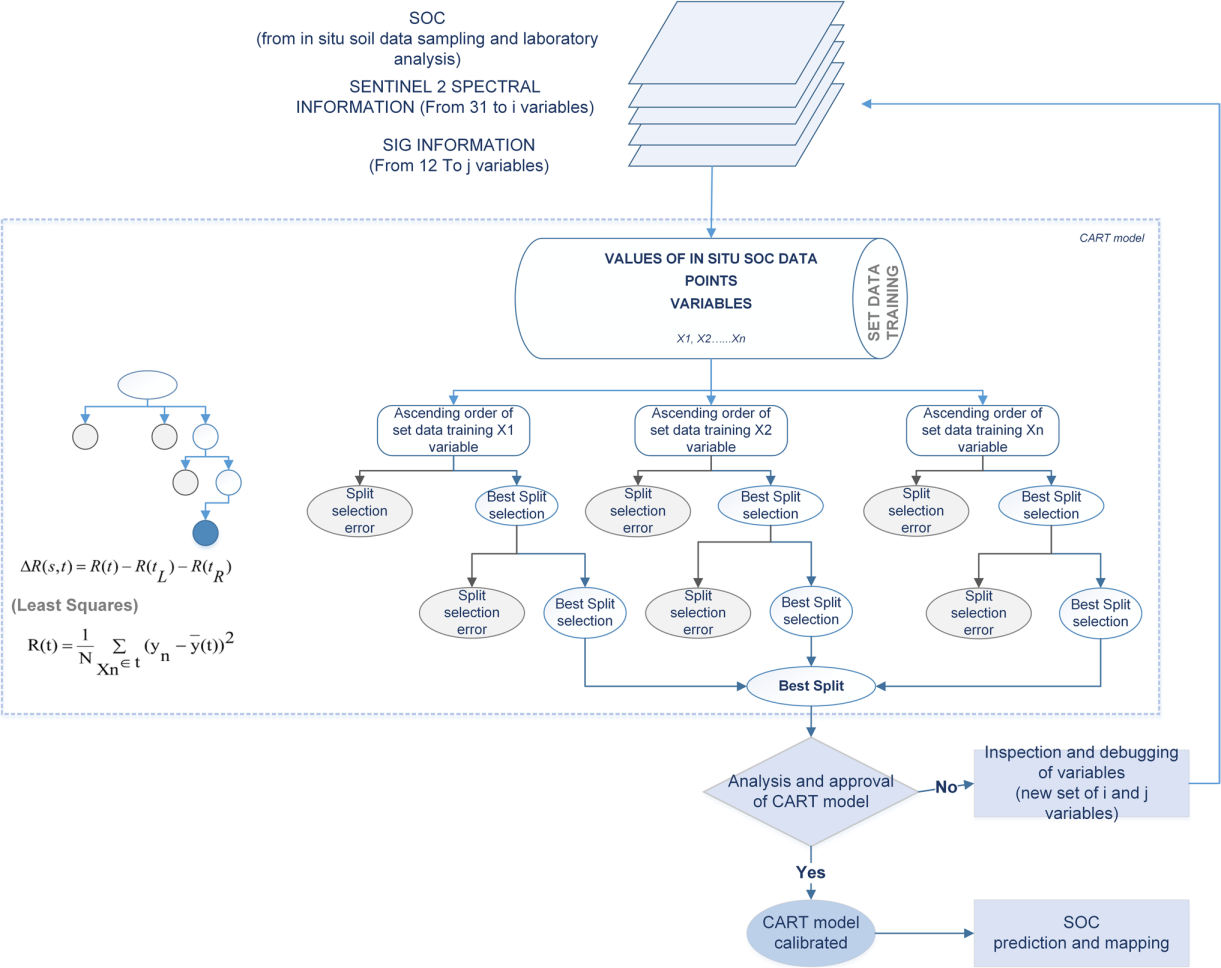
Methodology using CART model. Spliting and growing of CART tree was based on Breiman et al. (1984); Breiman (2001); and Salford Systems-Minitab Company (2017)

From 113 soil data samples and 43 variables selected, we calibrated a CART regression model using 80 soil data samples for training and 33 soil data samples for validation. The CART algorithm uses the data training set, which is composed of first the target variable (80 SOC data sampling), second variables to assess as SOC predictors (43 variables detailed in Table 2) to generate a tree. The root node is used to create decision trees, which have splits and end with end nodes. All variables are considered for each learning tree to find the best split. Each split refers to only one variable at once with a value or group of values, but the algorithm chooses the split with less error square and absolute error. The CART regression tree initiates its growth from a single root node. Once a node is split, it becomes a terminal node and cannot be further subdivided (Fig. 2).

**Table 2:** Environmental variables assessed as SOC bioindicators

Variable	Detail	Expression
Spectral bands from S2-MSI	*(S2 Band – central wavelength)* *B1-443 nm* *B2-490 nm* *B3-560 nm* *B4-665 nm* *B5-705 nm* *B6-740 nm* *B7-783 nm* *B8-842 nm* *B8a-865 nm* *B9-940 nm* *B10-1375 nm* *B11-1610 nm* *B12-2190 nm*	*S2-MSI bands* (ESA 2015) *B1* *B2* *B3* *B4* *B5* *B6* *B7* *B8* *B8a* *B9* *B10* *B11* *B12*
Spectral Indices derived from S2	*BI-* Bare Soil Index (Chen et al. 2004)	$BI=\frac{\left(\text{SWIR}1+\text{R}\right)-(\text{NIR}+\text{B})}{\left(\text{SWIR}1+\text{R}\right)+(\text{NIR}+\text{B})}$
	*EVI2-* Enhanced Vegetation Index 2 (Jiang et al. 2008)	$EVI2=2.5 \frac{\text{NIR}-\text{R}}{\text{NIR}+2.4\text{ R}+1}$
	*NBR-* Normalized Burn Ratio (Key and Benson 2006)	$NBR=\frac{\text{NIR}-\text{SWIR}2}{\text{NIR}+\text{SWIR}2}$
	*NBR2*- Normalized Burn Ratio 2 (Stokey et al. 2016)	$NBR2=\frac{\text{SWIR}1-\text{SWIR}2}{\text{SWIR}1+\text{SWIR}2}$
	*NDMI-* Normalized Difference Moisture Index (Wilson and Sader 2002; Hislop et al. 2018)	$NDMI=\frac{\text{NIR}-\text{SWIR}1}{\text{NIR}+\text{SWIR}1}$
	*NDSI-* Normalized Difference Snow Index (Takeuchi and Yasuoka 2004)	$NDSI=\frac{\text{SWIR}1-\text{NIR}}{\text{SWIR}1+\text{NIR}}$
	*NDVI-* Normalized Difference Vegetation Index (Rouse et al. 1974)	$NDVI=\frac{\text{NIR}-\text{R}}{\text{NIR}+\text{R}}$
	*NDWI-* Normalized Difference Water Index (McFeeters 1996)	$NDWI=\frac{\text{G}-\text{NIR}}{\text{G}+\text{NIR}}$
	*SAVI—*Soil-Adjusted Vegetation Index (Huete 1988), L value according to Ayala et al. (2017)	$SAVI=\frac{\text{NIR}-\text{R}}{\text{NIR}+\text{R}+\text{L}}\left(1+\text{L}\right)$ $\text{L}=0.15$
	*SeLI—*Sentinel-2 LAIgreen Index (SeLI) (Pasqualotto et al. 2019)	$SeLI=\frac{{\text{NIR}}_{{\text{R}}_{865}}-{\text{Red Edge}}_{{\text{R}}_{705}}}{{\text{NIR}}_{{\text{R}}_{865}}+{\text{Red Edge}}_{{\text{R}}_{705}}}$
	*VARI*_*g*_*—*Visible Atmospherically Resistant Vegetation Index green (Cammarano et al. 2014; Gitelson et al. 2002)	${\text{VARI}}_{\text{G}}=\frac{\text{G}-\text{R}}{\text{G}+\text{R}}$
	*WDRVI—*Wide Dynamic Range Vegetation Index – WDRVI (Gitelson 2004), a value according to Ayala et al. (2017)	$WDRVI=\frac{\text{aNIR}-\text{R}}{\text{aNIR}+\text{R}}$ $\text{a}=0.05$
Biophysical variables from S2	*FAPAR—*Fraction of Absorbed Photosynthetically Active Radiation FVC—Fraction of vegetation cover *LAI*—Leaf Area Index LCC/C_ab_—Chlorophyll content in the leaf *CWC*—Canopy Water Content (CWC)	S2 algorithms from SNAP (ESA 2024)
*LS Factor*	The topographic factor takes into account the influence of topography over soil erosion, based on length and slope inclination (Desmet and Govers 1996; Panagos et al. 2015; Lu et al. 2020).	$LS=\text{L}*\text{S}$ L factor A_i,j=_ accumulation area with coordinates (i,j) [m^2^]; D = pixel size [m]; x = shape coefficient [dimensionless]; m = values are between 0 and 1 [dimensionless]; $\uptheta$ = slope angle [rad]; and $\upbeta$ = ratio of rill to interill erosion [dimensionless] $\beta =\frac{\frac{sin\theta }{0.0896}}{3sin\;{\theta }^{0.8}+0.56}$ $m=\frac{\beta }{\beta +1}$ $L=\frac{{\left[{A}_{i,j}+D\right]}^{(m+1)}-{{A}_{i,j}}^{(m+1)}}{{x}^{m}{D}^{m+2}{(22.13)}^{m}}$ S factor If $tg\;\theta < 0.09$ then $S=10.8\mathit{sin}\;\theta +0.03$ else $S=16.8\mathit{sin}\;\theta -0.05$
*K Factor*	The K factor (K_Ru_sle) (Hengl et al. 2017): considers the susceptibility of the soil to erosion associated with properties such as texture, organic carbon content, type of structure and permeability (Miranda and Viloria 2020)	${K}_{Rusle}={f}_{csand}\times {f}_{clay-silt}\times {f}_{Orgc}\times {f}_{hisand}$ ${f}_{csand}=\left[\text{0,2}+\text{0,3}\times \mathit{exp}\left(-\text{0,256}{\times m}_{s}\times \left(1-\frac{{m}_{silt}}{100}\right)\right)\right]$ ${f}_{clay-silt}={\left(\frac{{m}_{silt}}{{m}_{c}+{m}_{silt}}\right)}^{\text{0,3}}$ ${f}_{hisand}=\left(1-\frac{0.70\times \left(1-\frac{{m}_{s}}{10}\right)}{\left(1-\frac{{m}_{s}}{100}\right)+\mathit{exp}\left[-\text{5,51}+22.9\times \left(1-\frac{{m}_{s}}{10}\right)\right]}\right)$ ${f}_{orgc}=\left(1-\frac{\text{0,25}\times orgC}{orgC+\mathit{exp}\left[\text{3,75}-\text{2,95}\times orgC\right]}\right)$ where: ${\text{m}}_{\text{s}}:$ sand fraction content percentage (0.5 to 2 mm particle diameter) [%] ${\text{m}}_{\text{silt}}$**:** percentage of silt fraction content (0.002 to 0.05 mm particle diameter) [%] $\text{mc}$: clay fraction content percentage (< 0.002 mm particle diameter) [%] $\text{orgC}$**:** organic carbon fraction content percentage [%]
*Texture-Clay*	15–30 cm	Geographical information from Ecuador (Ministerio de Agricultura y Ganadería-MAG 2023)
*Sand* (Soil sand content)	15–30 cm [%]	
Silt	15–30 cm [%]	
*Elevation*	From digital elevation model (DEM) [m a.s.l]	National information system of Ecuador (SNI 2011)
*Slope*	in [◦]	Obtained from DEM
*Flow direction*	Creates a raster of flow direction from each cell to its downslope neighbor, or neighbors, using D8, Multiple Flow Direction (MFD)	
*Flow accumulation*	Calculated from DEM	
*Precipitation* (Annual precipitation)	in [mm] of accumulation	(Funk et al. 2014; CONAE 2023)
*Temperature*	Annual average in [◦]	(University of Seville Climate Research Group 2023)
*Geological Unit*	Categorical variable based on the origin and evolution of soils over time	(Ministerio de Agricultura y Ganadería-MAG 2023)
*Taxonomic suborder*	Categorical variable based on taxonomic suborder of soil	
*Protected area*	Categorical variable to identify protected areas

The model is calibrated and validated after probing multiple alternatives. When the indicator capacity of some variables does not show a relative importance to the model, i.e., its information does not explain the SOC variability, then these variables are eliminated (Fig. 2). The model is optimized when the number of variables is minimized, achieving a high determination coefficient (R^2^) (Eq. 2) and the Root-Mean-Square Error (RMSE) is stable (Eq. 3) using a validation data set (33 SOC data sampling). The Absolute Deviation (MAD) (Eq. 4) and Mean Absolute Percentage Error (MAPE) (Eq. 5) were also analyzed:

2 $${R}^{2}=1-\frac{\sum_{i}^{n}{\left({y}_{i}-{f}_{i}\right)}^{2}}{\sum_{i}^{n}{\left({y}_{i}-\overline{y }\right)}^{2}}$$

3 $$RMSE=\sqrt{\sum_{i=1}^{n}{\frac{\left({f}_{i}-{y}_{i}\right)}{n}}^{2}}$$

4 $$MAD=\frac{\sum_{i}^{n}{|y}_{i}-{f}_{i}|}{n}$$

5 $$MAPE=\frac{100}{n}\sum_{i}^{n}\frac{{|y}_{i}-{f}_{i}|}{{y}_{i}}$$

where y_i_ is the observed value; f_i_ is the predicted value; $\overline{y }$ is the mean of the observed data; and n is the number of sample points.

## Results

**Fig. 3:**
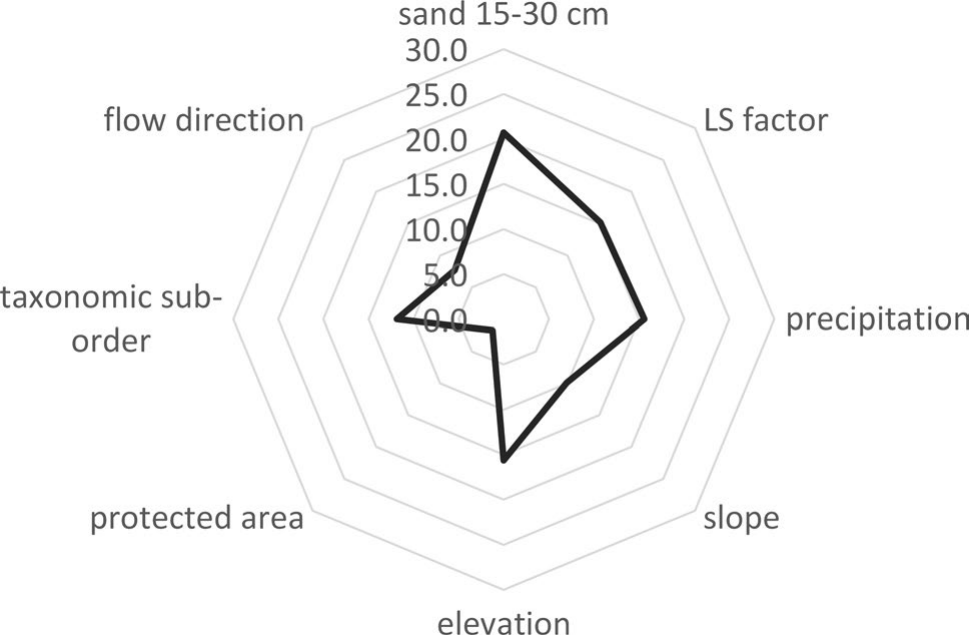
Relative importance of SOC predictors in the calibrated model for SOC in [%]. The sum of the relative importance of all predictors corresponds to 100% of the CART model

**Fig. 4:**
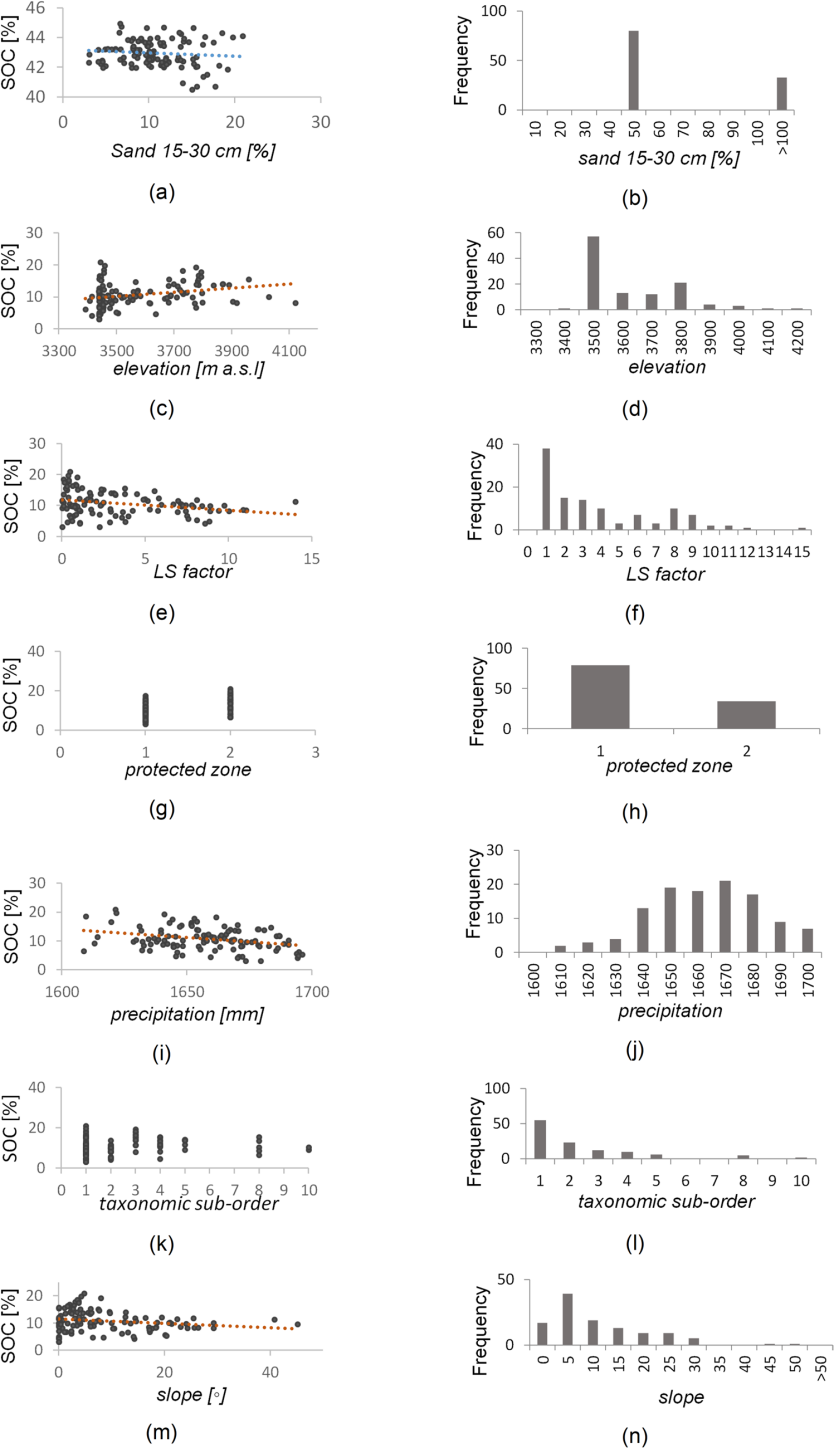
Scatterplots of SOC predictors vs. SOC data from in situ sampling; the trend line is shown (left). Histograms for the SOC predictors found (right)

The goodness-of-fit results against the validation dataset yielded a value of R^2^ of 0.67 for SOC in [%], and 0.34 for SOC in [Mg C ha^−1^], whereas the RMSE was 2.17 for SOC in [%] and 60.61 for SOC in [Mg C ha^−1^] (Table 3). The results for SOC in Mg C ha^−1^ revealed a need to increase the number of SOC data samples in the training model. The model for SOC in [%] allowed us to identify eight key SOC predictors with different significance levels. Predictors in the model for SOC % ordered by importance were sand 15–30 cm > precipitation > LS factor > elevation > taxonomic suborder > slope > flow direction > protected area (Fig. 3). A comparative analysis of in situ SOC data sampling against the values of each predictor was performed separately from the model (Figs. 2 and 4). Some variables showed relationships with SOC variables as a result of multiple calibration CART algorithms. These variables were removed due to their low relative importance in the model. However, the group of identified variables worked together and provided information linking with the SOC spatial dynamic in the study area.

**Table 3:** Model error measurements for SOC are shown as % and Mg C ha^−1^

%Error	SOC [%]	SOC [Mg C ha^−1^]
RMSE	2.17	60.61
MAD	1.21	39.98
MAPE	13.51	23.36
R^2^	0.67	0.35

The trend line revealed that an increase in sand content can cause a slight decrease in SOC (Fig. 4a), and the majority of the soils show a sand percentage around 50% (Fig. 4b). Soil samples were collected at elevations from approximately 3400 to 4200 m a.s.l.; this range of elevation values compared with SOC values showed a pattern of increasing SOC with increasing elevation (Fig. 4c, d). The LS Factor shows a decreasing trend when this erosion factor increases, which leads to decreasing SOC values. The high values of LS factor indicated that there were some areas with erosion processes in the study area (Fig. 4e, f). Regarding precipitation, this predictor ranged between 1600 and 1700 mm, and the trend followed by the SOC values showed a slight decrease with higher precipitation values (Fig. 4i, j). Slopes do not exceed 30º for the majority of the study area, with the steepest slopes corresponding to ravines. For these low slopes, the results showed a high variability in SOC values in comparison with higher slopes (Fig. 4m, n). Based on DEM values with cell sizes of 30 × 30 m and on the determined slopes, flow direction determined the water direction from one cell to one or more of its adjacent cells, yielding categorical values (i.e., north-64, south-4, east-1, west-16, northeast-128, northwest-32, southeast-2, and southwest-8). The results revealed a water flow in the northeast and southwest directions. Protected areas and taxonomic suborders are categorical predictors. The results revealed that the protected area (categorical value of 2) of each micro-watershed has a higher SOC range than the unprotected area (categorical value of 1). SOC values also varied as a function of taxonomic suborders.

**Fig. 5:**
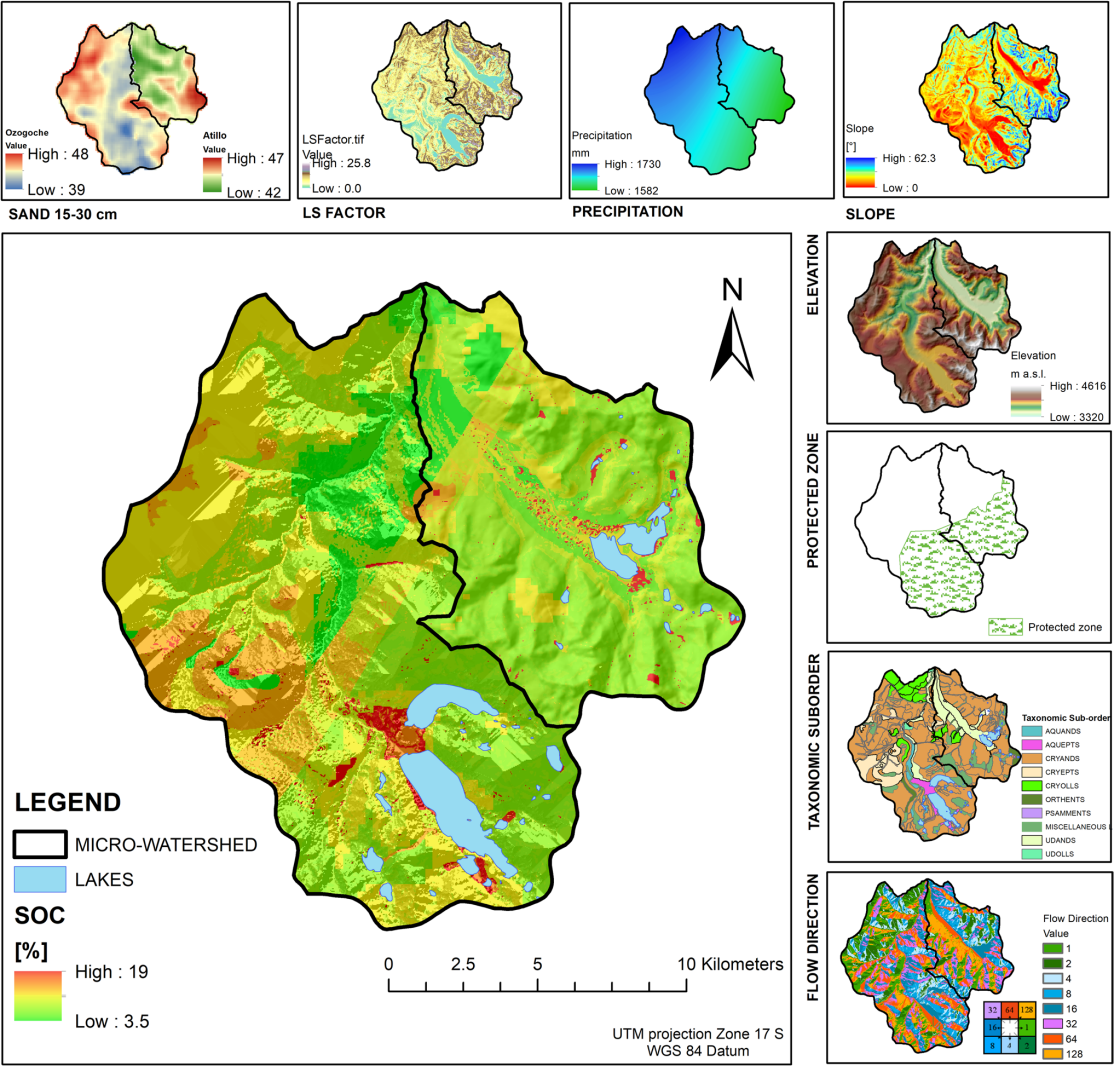
Predicted SOC % (0–30 cm) in the Atillo and Ozogoche micro-watersheds, using the CART model

Regarding SOC mapping type using the developed models, Fig. 5 displays the SOC% distribution in Atillo and Ozogoche micro-watersheds. Due to the low ${R}^{2}$ obtained for SOC in Mg C ha^−1^, SOC was not mapped in this unit. These results revealed a high SOC storage capacity, which was increased in the lake surroundings. SOC values ranging from 3.6% to 20.3% for SOC%, were revealed for the Atillo and Ozogoche micro-watersheds. SOC data sampling was not possible in the rocky areas with the highest elevation due to their extreme inaccessibility (Fig. 1); hence, uncertainty in the model would occur in the south part of Atillo and in the west part of Ozogoche.

**Fig. 6:**
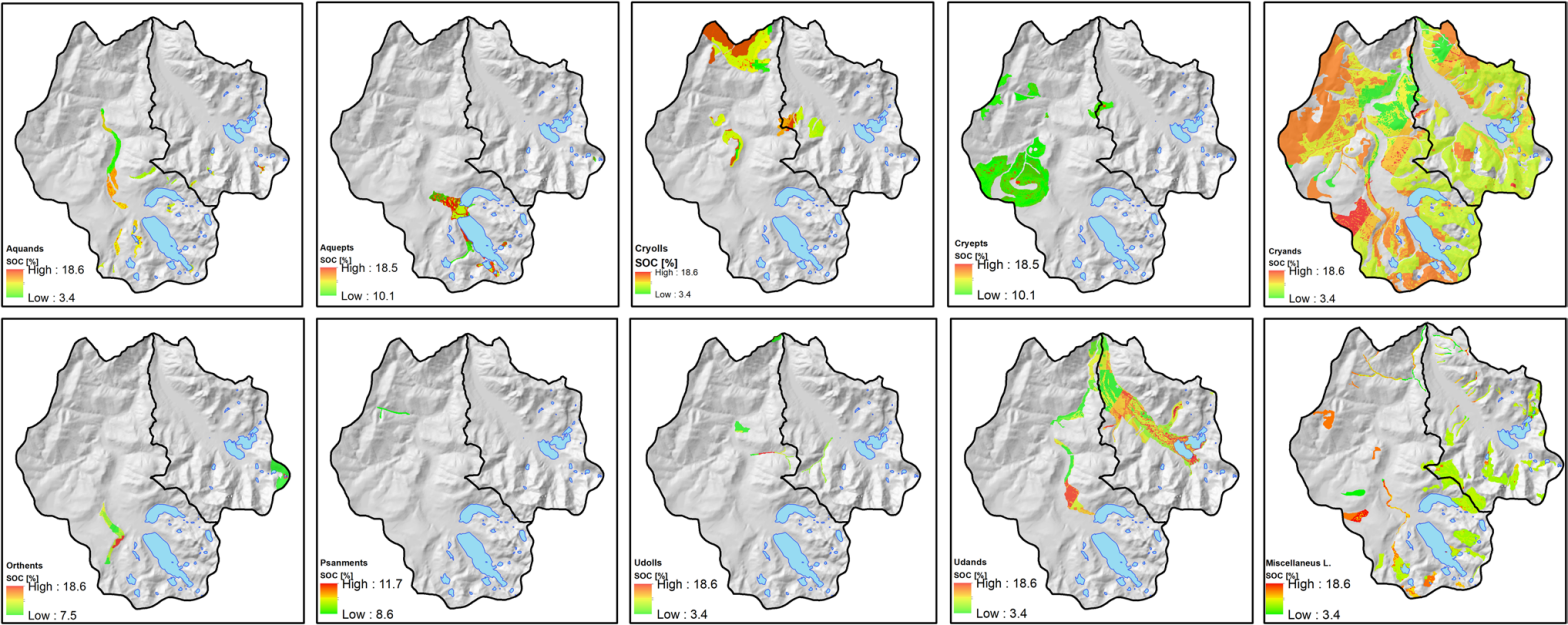
SOC [%] contents by soil taxonomic sub-order

With respect to taxonomic suborders of soils, Aquepts showed a high SOC %, which can be explained due to their adjacency to the lakes (Fig. 6 and Table 4). Conversely, Udolls showed the lowest SOC% with a mean of 7.3%. The majority of the area corresponds to Cryand soil type; it covers approximately 62%, with a mean SOC% around 9.1%. Differences between SOC value ranges of soils are observed in the minimum values, with values ranging from 3.4 to 10.1% where Aquepts and Cryepts are shown 10.1% of SOC.

**Table 4:** Statistics by soil taxonomic sub-order

Taxonomic sub-order	Area [ha]	MEAN [%]	DevSt
Aquepts	457.34	15.5	3.18
Cryepts	2067.37	13.5	1.2
Psanments	33.41	10.3	1.6
Orthents	384.38	9.9	3.3
Aquands	607.36	9.4	3.6
Cryolls	1751.02	9.4	2
Cryands	17,469.52	9.1	2.4
Miscellaneous Lands	2657.77	8.6	2.2
Udands	2522.76	8.5	3.7
Udolls	181.63	7.3	3.9

## Discussion

### Granulometric fractions and their influence on the SOC reserve or storage

Sand content showed plays an important role for the SOC model; soils in the study area had a sand content ranging from 42 to 47% in the Atillo micro-watershed and from 39 to 48% in Ozogoche. It is an important characteristic for SOC distribution, mainly in the uppermost 30 cm depth. It can control the mechanical properties of soils and cause hydro-morphism and cryogenics for SOC sequestration, which is in agreement with Angst et al. (2023).

High percentages of SOC were observed in soils based on their taxonomic suborder, with minimum and maximum values of 7.3% and 15.5%, respectively. Generally, soils exceeding 5% SOC have excellent structure, high fertility, and strong water infiltration and retention capacities. According to Yang et al. (2016), SOC levels in grassland soils range from 0.38% to 5.32%. These soils are characterized by their sandy loam texture, as opposed to sandy croplands, which indicate reduced fertility with SOC contents lower than 1.9%. Soils in the study area have high sand percentages combined with high content of SOM. This situation leads to low bulk density values, between 0.6 and 0.8 g.cm^−3^, observed in sampling soils, and a maximum SOC concentration ranging from 13 to 14% (Beltrán-Dávalos et al. 2022).

The developed model shows an importance of 20.7% for the sand 15–30 cm variable. Therefore, changes in SOC were identified based on differences in sand percentages (Figs. 4 and 5). These results can explain previous results in high Andean grasslands, which revealed an increase in SOC at low sand contents (Ćirić et al. 2013; Devi 2021). In contrast, some places, such as the surroundings of lakes in the Atillo micro-watershed showed discrepancies in this pattern (Fig. 5), which aligns with findings by Ghimire et al. (2024), who found that sand promotes SOC accrual in soil orders. Páramo soils may exhibit an exception that could be explained by the role of SOM in maintaining the balance and stability of SOC, regardless of the size of the predominant soil fractions (Paz et al. 2016; Ledo et al. 2020).

Despite their high sand content, these soils have a high water retention capacity. This is attributed to the presence of SOM, in interaction with low temperatures, soil moisture, and endemic vegetation. These dynamics were observed in soils in the proximity of water bodies, where the persistent precipitation and low temperatures contribute to higher levels of soil moisture and, consequently, to higher SOC content compared with soils in other ecological systems (Yost and Hartemink 2019). However, anthropogenic activities can alter the hydro-physical soil properties of Andean páramos (Patiño et al. 2021), causing a loss of SOC accumulation.

### Analysis of topographic conditions

The developed model included the variable “LS factor”, which revealed the importance of erosion for SOC accumulation. According to Castañeda-Martín and Montes-Pulido, (2017), the herbaceous and bush páramo ecosystems in the Peruvian Andean paramo, located above 3300 m a.s.l. (Manu National Park Manu and North Yauyos Cochas National Park, respectively), can accumulate SOC contents from 119 up to 397 t/ha in the upper 40 cm of soil. Soil erosion and deposition stabilize at least 0.72 Pg of carbon per year globally, and at the watershed level, this corresponds to approximately 16% of the eroded carbon (Berhe et al. 2007). This makes it a variable of interest due to the mobilization of SOC from one location to another. Moreover, up to 85% of the causes are related to grassland and agricultural land uses (Nadeu et al. 2010) where soil particles are moved (i.e., SOM) from high to low areas.

Landscape-scale interactions between erosion processes, weather patterns, vegetation cover, and land-use practices collectively account for approximately 67% of the observed variability in SOC distribution. This relationship has been modeled using combined techniques (Padarian et al. 2020). Soil erosion occurs naturally in all climatic conditions but is significantly increased and accelerated by human activities (FAO 2016). In addition, erosion levels can affect the sensitivity of an ecosystem. Hence, a considerable proportion of SOC is classified as labile, accentuating mineralization processes and methane generation in anaerobic soils. Emissions associated with erosion can reach between 800 and 1200 of t per year, which has a significant impact on the global carbon cycle (FAO 2016).

The spatial distribution of SOC can be affected by the hydric erosion process in Andean ecosystems (Calderón-Medina et al. 2018). The results of this study identified the influence of topography as a key variable in the model, which can be observed in the spatial distribution and redistribution of SOC (Figs. 4 and 5). Plains and hills revealed more SOM and subsequently more SOC, also reaching values over 100 Mg C ha^−1^ below the ground (Adhikari and Hartemink 2016), which are consistent with the results of this study. Mountain soils have a rather low SOC content (Adhikari and Hartemink 2016), which can be explained by elevation-related variables; soil thickness decreases and stoniness would be in favor of obtaining glacier conditions, affecting SOC storage, which is more evidenced in Fig. 5, where areas with low SOC values are related to the highest elevations.

The slope was a key factor determining SOC concentration in this study, which is in concordance with (Madrigal Reyes et al. 2019). The importance of this variable could be related to its change related to variations in land uses that can alter SOC content (Jakšić et al. 2021). Slopes lower than 15° show greater SOC content in comparison with slopes between 15° and 35° (Yu et al. 2020). Therefore, the slope variable is important not only to local but also to global SOC cycles. Effective soil conservation can mitigate risks and contribute to C sequestration in soil and biota (Zamora-Morales et al. 2018). Hence, it is important to implement policies aimed at preventing erosion in hilly areas with slopes greater than 35°, as well as in valleys, lakes, and rivers, where significant carbon transfers occur (Yu et al. 2020).

In addition, the highest SOC concentrations were predominantly found in the middle and lower areas of the Atillo and Ozogoche micro-watersheds. However, SOC alterations could be closely related to altitude. It can be explained by the influence of the elevation variable on the vegetation and biomass present at different elevation ranges (Zhang et al. 2021). Our results report greater SOC concentrations at 3200 to 3600 m a.s.l., which is typical of mountain herbaceous ecosystems at a constant height (Zhang et al. 2020). Contrasting results were obtained in regions over 3800 m a.s.l., where SOC values were lower. This is apparently caused by the evident rejuvenation of soil that would be induced by erosive processes, low temperatures, and events derived from the precipitation process. These conditions, in addition to the presence of endemic vegetation from the herbaceous páramo and peatlands, cause a significant increase in the absorption capacity of organic carbon in soils (Zhang et al. 2021).

Regarding flow direction, values were collected using the method known as D8. In this method, flow direction is determined by the direction of steepest descent, or maximum drop, in each cell (i.e., North-64, South-4, East-1, West-16, Northeast-128, Southeast-2, Northwest-32, and Southwest-8) (Jenson and Domingue 1988). We observed that flows come from the northeast to the west. Therefore, the presence of hydric accumulation and settleable materials is expected to occur in the Atillo and Ozogoche rivers. These rivers flow into the Cebadas river and subsequently form the hydric watershed of the Chambo river, which in turn flows into the Pastaza river, located in the Amazonian region of Ecuador (GAD provincia de Chimborazo 2015). Given that the studied micro-watersheds are in the Real range and were formed between the Jurassic and Pliocene periods, their geology is favorable for SOC storage. Therefore, the results highlight the environmental interest of research activities in the Real mountain range (Villamarín et al. 2014). Our results are also consistent with those of Ayala Izurieta et al. (2021) and Zermeño-González et al. (2011), underscoring the fact that the Real mountain range hosts a great diversity of ecosystems with the largest páramo areas in the world (Humerez and Umeda 2013).

### Influence of climatic conditions

The importance of climatic conditions for SOC sequestration has been identified, mainly in soils corresponding to the high Andean areas, where precipitation and temperature are considered determining parameters for its accumulation (Ayala-Niño et al. 2018; Ayala Izurieta et al. 2021, 2022). SOC storage and flow had shown low levels related to CO_2_ retention under weather conditions with low precipitation and high temperatures (Arriaga and Maya 2007). Low precipitation influences the growth of plant species, which are an insufficient source for biomass production. Moreover, the low fertility in semi-arid ecosystems, where the mineralization processes of organic compounds are higher, leads to lower SOC sequestration (Arriaga and Maya 2007).

SOC storage in regions with lower elevation, such as the Paraguaná Peninsula, have reported values between 7.8 and 101.5 Mg.ha^−1^ (Mogollón et al. 2015), whereas the SOC values in Atillo and Ozogoche where the accumulated precipitation is between 1600 and 1700 mm per year, reach approximately 451 Mg C ha^−1^. These values are higher than those reported by Vallejo et al. (2005) in an Andean zone, with reported SOC values below 337.98 Mg C ha^−1^. This Andean mountain ecosystem reveals its high capacity for SOC storage, influenced by its low temperatures and high precipitation depending on the elevation gradient (Sánchez et al. 2005).

A uniform rainfall regime was observed from 2002 to 2021, with average monthly rainfall ranging from 55 to 154 mm in Atillo, and 53 mm to 151 mm in Ozogoche. This characteristic is typical of the Herbaceus páramo ecosystem. The low temperatures and precipitation combined with the fog generate humid conditions throughout the year. This influences the formation of SOM, retention, and infiltration of water, highlighting the formation of carbon reserves and water resources (Huamán-Carrión et al. 2021). Hence, weather conditions play a main role in the dynamic of the SOC cycle in tropical areas (Saynes et al. 2005; Craine 2009; Campo et al. 2016), through which the health and sensitivity of soils can also be interconnected (Orjuela 2016).

### Model for SOC % vs model for SOC in Mg C ha.^−1^

SOC percentage is considered a relative variable that reveals the amount of SOM in the soil. SOC expressed in Mg C ha^−1^ is calculated taking into account bulk density, which is an important indicator for assessing the productivity of an ecosystem (Calderón-Medina et al. 2018). The apparent density is influenced by indicators that include the amount of sand, precipitation, and slope. The high variability in the contributions of SOC is associated with high percentages of peat material or grass tissues. This causes a decrease in bulk density, as occurring in high Andean ecosystems in Colombia and Peru (Abarca and Galdos 2020) with ratings lower than 0.6 g/cm^3^. Measurements of SOC expressed in Mg C ha^−1^ refer to an absolute measure of the total amount of SOC, which would be more realistically considered in the analyzed properties and functions of soils as ecosystem service providers (Pereira et al. 2018). Nevertheless, our study reported a decrease in R^2^ for the SOC model expressed in Mg C ha^−1^ compared with the model expressed in %. These findings align with the results from the páramo region in the central zone of Ecuador, where R^2^ values of 0.82 for SOC in % and 0.77 for SOC in Mg C ha^−1^ were reported (Ayala Izurieta et al. 2022), indicating a decrease of 0.05 in R^2^ for SOC in Mg C ha^−1^ relative to SOC%. This reduction is attributed to the influence of bulk density. In contrast to the latter study, variables identified as SOC predictors in more extensive study areas, such as Geological Unities and Soils Taxonomy, exhibited diminished influence within the present model. However, our study showed a reduction of R^2^ value of only 0.15 for SOC% due to the new SOC predictors (i.e., sand 15–30, taxonomic suborder, slope > flow direction > protected area).

After assessing 43 variables with multiple CART algorithms, eight key variables were found to govern the model. Sand was the main driving variable to explain SOC changes, with a 20.7% importance, while the variables LS factor, precipitation, and elevation showed an importance over 15%. The importance of the taxonomic sub-order variable was 11.9%, lower than expected given that taxonomic-suborder is dependent on soil taxonomy. Due to the complex spatial distribution, inclusions of Andosols had been previously reported as Entisols (Ayala Izurieta et al. 2022). Therefore, our results suggest that “soil taxonomy” variable could have a reduced efficiency in model performance.

Our study also reveals the importance of assessing new variables as SOC predictors within reduced areas to maximize heterogeneity. New driving variables to explain SOC variability were obtained (i.e., sand 15–30 cm, taxonomic suborder, flow direction, protected area), which promote the growth of learning trees and facilitate the tree splitting process. Follow-up studies could focus on model refinement. A stratified sampling approach, integrating the SOC predictors identified in this study and using different machine learning algorithms, can be applied to enhance accuracy.

Finally, SOC dynamics in soil are complex and exhibit strong spatial and temporal variability (Sleutel et al. 2006). Páramo highlands extend from Venezuela to Peru, with two additional, more isolated complexes in Central America (Hofstede et al. 2014). Therefore, our study can be replicated in other páramo regions and in other regions with similar conditions. Further local-scale studies are necessary since local governments in rural areas are responsible for caring for and directly administering the zone; moreover, areas are delimited mainly by hydrology, such as micro-watersheds. The results of this study can be used to establish new strategies for the preservation and conservation of ecosystems and protected areas. This is due to the fact that land use clearly affects soil conditions (Poulenard et al. 2001). For instance, anthropogenic activities can lead to a loss of SOC storage.

## Conclusions

High Andean páramo soils exhibited a substantial capacity for SOC storage. This is explained by the low temperatures typical of this type of ecosystem, which decrease the SOM decomposition ratio in soils, which in turn promotes SOC accumulation. Therefore, understanding the spatial distribution of SOC is crucial for sustainable soil management in this region.

The predictive model for SOC % in the 0–30 cm soil profile achieved an accuracy of 67%. It was developed using geophysical, meteorological, topographic, and satellite data to calibrate algorithms based on CART regression trees. The accuracy of the SOC model worsened when expressing predictions in Mg C ha^−1^. This can be explained by the use of bulk density obtained by soil sampling, which is introduced into the calculation of SOC before training the model. This also suggests the need for rigorous soil data sampling to increase the accuracy of the model in Mg C ha^−1^. Moreover, the complexity of the area limits access to some sectors. Therefore, the model of SOC expressed in % is an alternative to assess these types of ecosystems and their soils. Since study area has a high capacity for SOC storage, preservation and conservation of these ecosystems can be considered a priority in undertaking efforts to reduce negative environmental impacts.

The SOC% model using CART regression trees allows us to identify eight SOC predictors: *sand 15–30 cm* > *Precipitation* > *LS Factor* > *elevation* > *taxonomic suborder* > *flow direction* > *Slope* > *protected area*, in order of importance (SOC in %).

SOC expressed in Mg C ha^−1^ is obtained using soil bulk density; these values have been influenced by the sand, precipitation, and slope variables. It suggests that SOC in Mg C ha^−1^ refers to an absolute and more realistic understanding of the amount of SOC. The assessment of SOC dynamics using variables extracted from S2 data, in conjunction with global and national mapping initiatives, enables the identification of two alternative ways to implement effective soil management strategies. The first one focuses on assessing the areas of greatest productivity, characterized by high percentages of SOC directly associated with SOM, more than 11% in the Real range system. This facilitates the identification and prioritization of vulnerable areas suitable for sustainable land use. The second way focuses on the estimation of ecosystem carbon sequestration potential. This estimation incorporates various soil parameters, including bulk density and soil profile characteristics. This allows assessing the ecological interest of these ecosystems and recognizing them as powerful soil CO_2_ storage systems. This contribution is useful for regulation and support services, prioritizing the conservation of high Andean areas in the Cordillera Real and promoting the creation and consolidation of buffer areas between agricultural lands and the National Reserve of the Sangay National Park.

## Data Availability

Data will be made available on request.
